# Supplementary material for: Diet Quality at 3 Years of Age Relates to Lower Body Mass Index but Not Lower Blood Pressure at 10 Years of Age
Source: Nutrients. 2024 Aug 9;16(16):2634. doi: 10.3390/nu16162634 (PMC11356893; doi:10.3390/nu16162634)
Supplement: Supplementary file 1 [file nutrients-16-02634-s001.zip › nutrients-3115688-supplementary.pdf]

Diet quality at 3 years of age relates to lower BMI but not lower blood pressure at 10 years of age

Qihua Wang [1,2] , Tian Xie [1], Xia Huo [2], Harold Snieder [1], Eva Corpeleijn [1,*]

[1] Department of Epidemiology, University Medical Center Groningen, University of Groningen, 9713 GZ Groningen, the Netherlands.

[2] Laboratory of Environmental Medicine and Developmental Toxicology, Guangdong Key Laboratory of Environmental Pollution and Health, College of Environment and Climate, Jinan University, Guangzhou 511443, China.

* Correspondence to:

Eva Corpeleijn, Department of Epidemiology, University of Groningen, University Medical Center Groningen, 9713 GZ Groningen, the Netherlands. Email: e.corpeleijn@umcg.nl

Supplemental Material

Table S1. Food groups of the 3 diet scores

Figure S1. Flowchart detailing participant inclusion and exclusion

Table S1. Food groups of the 3 diet scores

DASH			MDS			LLDS		
Food groups	Unit of daily intake	Values (points) 0 to 32	Food groups	Unit of daily intake	Values (points) 0 to 7	Food groups	Unit of daily intake	Values (points) 0 to 44
1 Vegetables	servings/ day	0 to 4	1 Fruit and nut	grams/ 1000 kcal	0, 1	1 Vegetables	grams/ 1000 kcal	0 to 4
2 Fruits		0 to 4	2 Vegetables and legumes		0, 1	2 Fruit		0 to 4
3 Whole grains		0 to 4	3 Potatoes and cereal grains		0, 1	3 Whole-grain products		0 to 4
4 Nuts and legumes		0 to 4	4 Fish products		0, 1	4 Legumes and nuts		0 to 4
5 Low fat dairy products		0 to 4	5 Dairy products		1, 0	5 Fish		0 to 4
6 Red and processed meat		4 to 0	6 Meat products		1, 0	6 Oils and soft margarines		0 to 4
7 Sugar-sweetened beverages		4 to 0	7 Unsaturated: saturated fats		0, 1	7 Unsweetened dairy		0 to 4

8	Sodium	mg/day	4 to 0

8	Tea		0 to 4
9	Red and processed meat		4 to 0
10	Hard margarines		4 to 0
11	Sugar-sweetened beverages		4 to 0

Vegetables = Vegetables without potatoes. Green labeled: Food groups with positive health effects. Red labeled: Food groups with negative health effects. DASH, Dietary Approaches to Stop Hypertension; MDS, Mediterranean Diet Score;LLDS, Lifelines Diet Score.

The daily intakes of each food product were calculated from FFQ data after verification and validation: Amount in portions × weighting value/sum of weighting values × correction factor × frequency per week/7 = daily intake in grams per day. A detailed list of food products in each food group of DASH, MDS, and LLDS can be found in our references [13, 59, 60]. Energy intake for each food product is needed to transfer the daily intake in grams to in grams/ 1000 kcal, whereas intakes of specific nutrients are needed for different diet scores, including sodium amount in mg/day for DASH, and unsaturated to saturated fats ratio for MDS. The energy and nutrient amounts of each unit of each food product were calculated from NEVO 2011. For DASH, the amounts of daily intake and sodium of each food product were summed up to the daily intakes of food groups (servings/day) and sodium (mg/day), and then ranked into quintiles and given positive or reverse scores. For food groups with positive health effects, quintile 1 to 5 were valued as 0 to 4. For food groups with negative health effects and sodium, quintile 1 to 5 were valued as 4 to 0. The scores of each food group

were summed up to a total score that ranged from 0 to 32. A higher DASH score represents better diet quality. For MDS, the daily intakes of each food group were expressed as a dichotomous ranking. High adherence was valued as 1, while low adherence was valued as 0, summing up to a total score that ranged from 0 to 7. A higher MDS represents better diet quality. High adherence to MDS was defined as an MDS higher than 3. For LLDS, the daily intakes of each food group were expressed in a quintile ranking of the sum of daily intakes of each product. For food groups with positive health effects, quintile 1 to 5 were valued as 0 to 4. For food groups with negative health effects, quintile 1 to 5 were valued as 4 to 0. The scores of 11 instead of 12 food groups (coffee excluded) summed up to a total score ranging from 0 to 44. Higher LLDS values represent better diet quality.

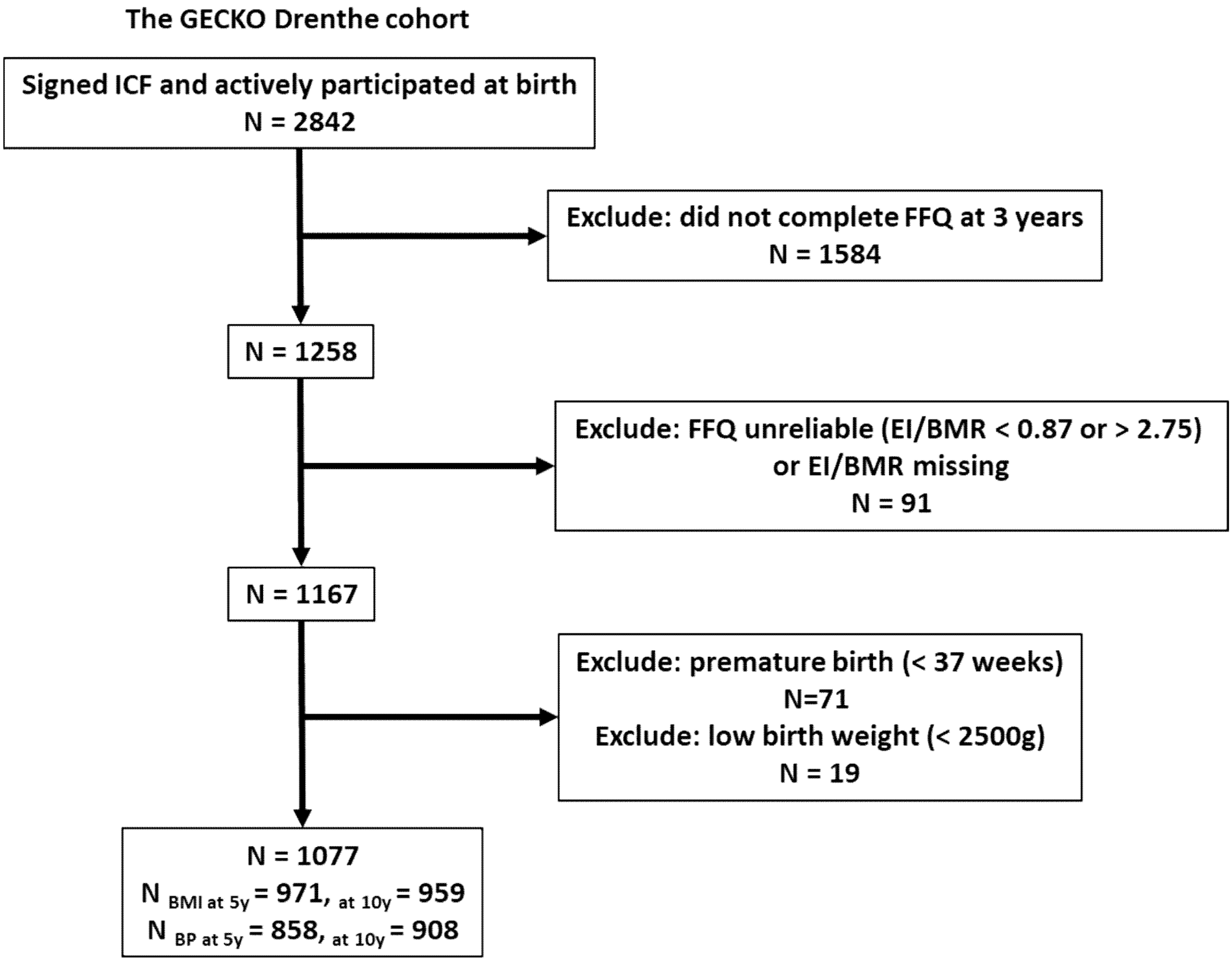

Figure S1. Flowchart detailing participant inclusion and exclusion

ICF: informed consent form. Actively participated: at least 1 questionnaire answered by the parents and/or available child measurements. FFQ: food frequency questionnaire. EI/BMR: energy intake/basal metabolic rate ratio. There are final 1077 individuals enrolled in the present study. Since the percentage of missingness was very low (all ≤ 3.1%), simple imputation by median or mode was used for the missing covariates. In BMI models, the percentages of missingness before imputation were 3.1% and 2.9% for paternal education level at 5 and 10

years respectively, and 0.1% for any smoking during pregnancy. In BP models, the percentages of missingness before imputation were 0.8% for height at 5 years, 1.2% and 0.8% for maternal education level at 5 and 10 years respectively, and 0.1% for any smoking during pregnancy.
